# Feature selection for elderly faller classification based on wearable sensors

**DOI:** 10.1186/s12984-017-0255-9

**Published:** 2017-05-30

**Authors:** Jennifer Howcroft, Jonathan Kofman, Edward D. Lemaire

**Affiliations:** 1 Department of Systems Design Engineering, University of Waterloo, Waterloo, Canada; 2 Centre for Rehabilitation, Research and Development, Ottawa Hospital Research Institute, Ottawa, Canada; 3 Faculty of Medicine, University of Ottawa, Ottawa, Canada

**Keywords:** Wearable sensors, Accelerometer, Plantar pressure, Fall risk, Older adults, Prediction, Faller classification, Feature selection

## Abstract

**Background:**

Wearable sensors can be used to derive numerous gait pattern features for elderly fall risk and faller classification; however, an appropriate feature set is required to avoid high computational costs and the inclusion of irrelevant features. The objectives of this study were to identify and evaluate smaller feature sets for faller classification from large feature sets derived from wearable accelerometer and pressure-sensing insole gait data.

**Methods:**

A convenience sample of 100 older adults (75.5 ± 6.7 years; 76 non-fallers, 24 fallers based on 6 month retrospective fall occurrence) walked 7.62 m while wearing pressure-sensing insoles and tri-axial accelerometers at the head, pelvis, left and right shanks. Feature selection was performed using correlation-based feature selection (CFS), fast correlation based filter (FCBF), and Relief-F algorithms. Faller classification was performed using multi-layer perceptron neural network, naïve Bayesian, and support vector machine classifiers, with 75:25 single stratified holdout and repeated random sampling.

**Results:**

The best performing model was a support vector machine with 78% accuracy, 26% sensitivity, 95% specificity, 0.36 F1 score, and 0.31 MCC and one posterior pelvis accelerometer input feature (left acceleration standard deviation). The second best model achieved better sensitivity (44%) and used a support vector machine with 74% accuracy, 83% specificity, 0.44 F1 score, and 0.29 MCC. This model had ten input features: maximum, mean and standard deviation posterior acceleration; maximum, mean and standard deviation anterior acceleration; mean superior acceleration; and three impulse features. The best multi-sensor model sensitivity (56%) was achieved using posterior pelvis and both shank accelerometers and a naïve Bayesian classifier. The best single-sensor model sensitivity (41%) was achieved using the posterior pelvis accelerometer and a naïve Bayesian classifier.

**Conclusions:**

Feature selection provided models with smaller feature sets and improved faller classification compared to faller classification without feature selection. CFS and FCBF provided the best feature subset (one posterior pelvis accelerometer feature) for faller classification. However, better sensitivity was achieved by the second best model based on a Relief-F feature subset with three pressure-sensing insole features and seven head accelerometer features. Feature selection should be considered as an important step in faller classification using wearable sensors.

## Background

Wearable sensors can be used to assess gait and predict elderly fall risk, with varying degrees of success [1]. Numerous gait pattern features can be derived from a wearable sensor; however, an appropriate feature set is required to avoid high computational costs, “curse of dimensionality”, and irrelevant features [2, 3]. Reducing feature-space size reduces the risk of prediction-model overfitting and may improve classification performance [3, 4]. Even with these benefits, few fall - risk models in the literature employ feature-space size reduction techniques to improve classification performance.

Various techniques have been used to reduce the feature-space size before faller classification for wearable-sensor-based elderly fall risk applications. Factor analysis uses a statistical technique to examine variability between correlated features, and represents that variability as fewer factor variables; for example, Riva et al. [5] used this method to represent 24 features as seven factors. Principal component analysis (PCA) is similar to factor analysis but uses orthogonal transformation to represent features as linearly uncorrelated variables called principal components. PCA was used to represent 24 dynamic stability features with three principal components representing global gait pattern kinetics, global gait regularity, and stride time [6]. Sequential forward floating search algorithms start with an empty set (i.e., no features) and add features, starting with the best feature, until classification accuracy is maximized. Liu et al. [7] used this method to reduce feature-space size from 123 features to as few as three features. Forward wrapper feature selection techniques can take many forms. The technique used by Caby et al. [8] was similar to a sequential forward floating search algorithm, starting with an empty set and adding features to maximize classification performance. This method reduced feature-space size from 67 features to as few as one feature [8]. Only these few studies, which used wearable sensor-derived features for faller classification, reduced feature-space size before faller classification [5–8].

To reduce feature-space size, feature selection techniques are preferable to projection techniques (e.g. PCA) and compression techniques (e.g. information theory) because the original features are not altered [4]. Three main feature selection methods can be considered: filter, wrapper, and embedded. Filter methods focus on intrinsic data properties, with features scored on relevance [3, 4]. Wrapper methods are developed for a specific classification method and different feature subsets are tested with the chosen classifier to optimize performance [3, 4]. Wrapper methods can achieve better performance than filter methods but are computationally expensive and can result in overfitting [4]. Embedded methods are similar to wrapper methods but feature selection is built into the classifier construction, which reduces computational complexity compared to wrapper methods [4]. Caby et al. [8] used a wrapper feature selection method to reduce a wearable-sensor-based feature space before using an intelligent classifier for fall risk prediction. While the wrapper approach is valid, this method ties feature selection to a specific classifier, precluding feature subset evaluation across different classifiers. A classifier-independent, filter approach is preferred because it permits direct comparisons between different classifiers and different feature sets, including a full feature set.

The objectives of this study were to identify smaller feature sets for faller classification from large feature sets derived from wearable accelerometer and pressure-sensing insole gait data, and to evaluate faller classification performance of these feature sets with different classifiers. This study also evaluated whether feature selection would improve faller classification performance compared to classification without feature selection. Successful application of feature selection techniques to faller classification would improve the clinical applicability of fall risk prediction models by reducing assessment and analysis complexity.

## Methods

### Participants

A convenience sample of 100 people, 65 years or older, were recruited from the community (Table 1). Participants were identified as fallers if they reported at least one fall during the six months prior to study participation. Potential participants were excluded if they had a cognitive disorder (self-reported) or were unable to walk for six minutes without an assistive device. The University of Waterloo Research Ethics Committee approved the study and all participants gave informed written consent.

**Table 1:** Participant characteristics (mean ± standard deviation)

	Participants (#)	Age (years)	Height (cm)	Weight (kg)	6MWT (m)
Fallers	13 male, 11 female	76.3 ± 7.0	165.2 ± 10.3	71.9 ± 14.3	446.6 ± 101.4
Non Fallers	31 male, 45 female	75.2 ± 6.6	165.1 ± 9.9	73.1 ± 13.4	455.8 ± 102.4

### Protocol

Participants reported six month retrospective fall occurrence, age, and sex. Body weight and height were measured.

Pressure-sensing insoles (F-Scan 3000E, Tekscan, Boston, MA) were equilibrated using multi-point calibration (137.9, 275.8, 413.7 kPa), fit to the shoes, and calibrated. Accelerometers (X16-1C, Gulf Coast Data Concepts, Waveland, MS) were attached to the posterior head with a band, posterior pelvis with a belt, and lateral shank, just above the ankle, with a band. Plantar pressure data were collected at 120 Hz and accelerometer data at 50 Hz. Completion time and wearable sensor gait data were collected while participants completed a 7.62 m (25 ft) walk.

### Wearable sensor features

Plantar-pressure derived features were:

- Center of Pressure (CoP) path: Number, length, and duration of posterior deviations per stance. Number, lateral length, medial length, and duration of medial-lateral (ML) deviations. Anterior-posterior (AP) and ML coefficients of variation (CoV) for the stance phase CoP path.
- Temporal: Cadence, stride time, stance time, swing time, percent stance time, percent double support time, stride time symmetry index [9] between the left and right limbs, and CoV for stride time, stance time, and swing time.
- Impulse: Impulse variables were determined from the total force-time curve and normalized by body mass (Ns/kg) for: I1 (foot-strike to first peak), I2 (first peak to minimum), I3 (minimum to second peak), I4 (second peak to foot-off), I5 (foot-strike to minimum), I6 (minimum to foot-off), and I7 (foot-strike to foot-off).

All variables were calculated for each stride for the left and right limbs before calculating means and standard deviations across both limbs (i.e. left and right limb combined).

For each accelerometer location, the accelerometer derived features were:

- Descriptive statistics: Maximum, mean, and standard deviation of acceleration for the superior, inferior, anterior, posterior, right, and left axes.
- Temporal: Cadence and stride time.
- Fast Fourier Transform (FFT): Percentage of acceleration frequencies in the first quartile of an FFT frequency plot for vertical, AP, and ML axes.
- Ratio of even to odd harmonics (REOH): Proportion of the acceleration signal in phase with stride frequency. The harmonic ratio was calculated for vertical, AP, and ML axes as in [10].
- Maximum Lyapunov exponent (MLE): Average rate of expansion or contraction of the original trajectory in response to perturbations [11, 12], calculated for vertical, AP, and ML accelerations, as in [13].

For descriptive statistics and MLE parameters, acceleration data were filtered using a fifth order, low pass Butterworth filter with a 12.5 Hz cut-off frequency. Unfiltered acceleration data were used to calculate the FFT quartile and REOH.

### Feature selection

Filter feature selection methods were selected because feature subsets from each filter method could be evaluated using three different classifiers, which would not be possible with wrapper or embedded methods. Furthermore, filter methods reduce the computational cost and reduce the risk of overfitting [4]. Three filter feature selection methods were used: correlation-based feature selection (CFS), fast correlation based filter (FCBF), and Relief-F. CFS and FCBF both provide a minimum subset of features whereas Relief-F provides a ranking of features.

CFS is a supervised method that identifies a subset of features that are correlated with the class label (i.e. faller or non-faller) and uncorrelated with other parameters, and eliminates irrelevant and redundant features [3]. To identify the feature subset, CFS computes the subset’s heuristic measure of ‘merit’ based on pairwise correlations [14, 15].

FCBF is a supervised method that identifies predominant features for classification and eliminates redundant features. This method avoids pairwise correlation analysis between all relevant features, reducing computational complexity compared to CFS [16]. The feature subset is selected based on the symmetrical uncertainty [14].

Relief-F is a supervised method that weights the parameter’s relative strength, and eliminates less relevant features without eliminating redundant features [2, 14]. Relief-F is useful when evaluating parameters with interdependencies and noisy data sets [15]. The number of features to include in the Relief-F feature subset was determined using the runExperiment algorithm within the Arizona State University Feature Selection Repository (ASUFSR) [14], which evaluates increasingly larger feature subsets, by five-feature increments, until the entire feature set is included in the subset. For the runExperiment algorithm, naïve Bayes (NB) and Support Vector Machine (SVM) were used as classifiers and a 75:25 stratified holdout was used for training and testing data division, which was also used for model development. The smallest feature subset that did not decrease overall accuracy, or at worst resulted in no more than a 5% decrease in accuracy from the full-feature set, was selected.

**Fig. 1:**
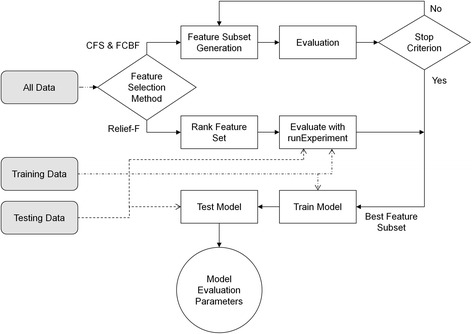
Flowchart of feature selection and model development process

Feature selection was performed (Fig. 1) on the entire dataset for all features for each of the 31 sensor combinations (Table 2) in Matlab R2010a using ASUFSR algorithms [14]. Because the entire dataset was used for feature selection, feature selection was not performed for each iteration of the prediction stability analysis. Identical feature subsets were used as inputs for both the single stratified holdout (model development) and all 10,000 randomized holdouts (prediction stability). Pelvis accelerometer data were missing for two non-fallers and left shank accelerometer data were missing for one non-faller due to sensor power failure.

**Table 2:** Sensor combinations and total number of input parameters (from [17], with permission from the publisher)

Sensor Combination	Sensor Description	Total parameters
I	pressure insole	30
H	accelerometer (head)	29
P	accelerometer (pelvis)	29
LS	accelerometer (left shank)	29
RS	accelerometer (right shank)	29
H-P	accelerometer (head, pelvis)	58
H-LS	accelerometer (head, left shank)	58
H-RS	accelerometer (head, right shank)	58
P-LS	accelerometer (pelvis, left shank)	58
P-RS	accelerometer (pelvis, right shank)	58
LS-RS	accelerometer (left shank, right shank)	58
H-P-LS	accelerometer (head, pelvis, left shank)	87
H-P-RS	accelerometer (head, pelvis, right shank)	87
H-LS-RS	accelerometer (head, left shank, right shank)	87
P-LS-RS	accelerometer (pelvis, left shank, right shank)	87
H-P-LS-RS	accelerometer (head, pelvis, left shank, right shank)	116
I-H	pressure insole; accelerometer (head)	59
I-P	pressure insole; accelerometer (pelvis)	59
I-LS	pressure insole; accelerometer (left shank)	59
I-RS	pressure insole; accelerometer (right shank)	59
I-H-P	pressure insole; accelerometer (head, pelvis)	88
I-H-LS	pressure insole; accelerometer (head, left shank)	88
I-H-RS	pressure insole; accelerometer (head, right shank)	88
I-P-LS	pressure insole; accelerometer (pelvis, left shank)	88
I-P-RS	pressure insole; accelerometer (pelvis, right shank)	88
I-LS-RS	pressure insole; accelerometer (left shank, right shank)	88
I-H-P-LS	pressure insole; accelerometer (head, pelvis, left shank)	117
I-H-P-RS	pressure insole; accelerometer (head, pelvis, right shank)	117
I-H-LS-RS	pressure insole; accelerometer (head, left shank, right shank)	117
I-P-LS-RS	pressure insole; accelerometer (pelvis, left shank, right shank)	117
I-H-P-LS-RS	pressure insole; accelerometer (head, pelvis, left shank, right shank)	146

*I* pressure-sensing insole measures, *H* head accelerometer measures, *P* pelvis accelerometer measures, *LS* left shank accelerometer measures, *RS* right shank accelerometer measures

### Model development

Following feature selection, three classifier models were used to assess each feature set: multi-layer perceptron neural network (NN) with 5 to 25 nodes in a single hidden layer, linear and quadratic discriminant NB, and SVM with one to seven degree polynomial kernels [17]. The classification criterion was retrospective fall occurrence. For all models, 75% of participant data (18 fallers, 57 non-fallers) were used for training and 25% were used for testing (6 fallers, 19 non-fallers). Faller classification using NN, NB, and SVM without feature selection was performed previously [17] using identical train:test groupings and input data, and the top ten models from that previous analysis were used in the current study to evaluate the effect of feature selection on classification performance.

**Fig. 2:**
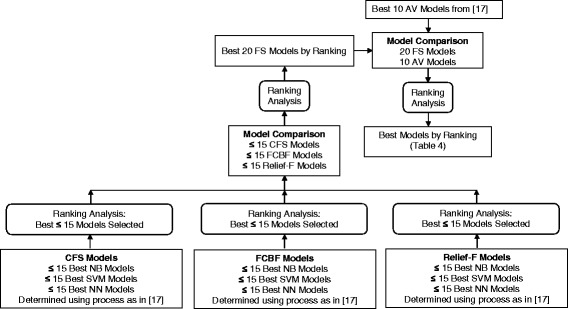
Flowchart of feature selection-based model development and ranking analysis. AV: All variable, FS: Feature selection, NB: Naïve Bayesian, NN: Neural network, SVM: Support vector machine.

Model evaluation parameters included accuracy, specificity, sensitivity, positive predictive value (PPV), negative predictive value (NPV) [18], F1 score (harmonic mean of precision and sensitivity) [19], and Matthew’s Correlation Coefficient (MCC) [20]. A ranking method similar to the approach used in Kendell et al., 2012 [21] was employed to determine the best models. Each model evaluation parameter was ranked from best (1) to worst (*n*), and ranks for all model evaluation parameters were summed to identify the overall best model (lowest summed rank) (Fig. 2). Confidence intervals (*CI*) for model accuracy were computed using the Wilson interval (Equation 1), which is an appropriate binomial proportion interval estimation method for small sample sizes [22].

1 $$ C I=\frac{p+\frac{z^2}{2 N}\pm z\sqrt{\frac{p}{N}-\frac{p^2}{N}+\frac{z^2}{4{N}^2}}}{1+\frac{z^2}{N}} $$

where *p* is the model accuracy, *N* is the number of participants in the test dataset, and *z* is 1.96 for a 95% confidence interval.

### Prediction stability

The prediction accuracy of the top twenty feature selection-based models and top ten all variable-based models were further examined by training 10,000 models with randomized 75:25 train:test stratified holdouts (repeated random sampling, RRS). Model evaluation parameters were calculated and averaged across all 10,000 models and then ranking was performed, as described previously.

## Results

Nine feature subsets (eight Relief-F, one CFS, one FCBF: Table 3) were inputs for the twenty best models (Table 4). CFS and FCBF analyses outputted the same feature set (Feature Subset 9). For the single 75:25 train:test stratified holdout, the top fifteen models used Relief-F feature selection, with the top two models (Feature Subset 1, SVM-6 and SVM-7) including three insole measures and seven head accelerometer measures (Table 3). The top model (Feature Subset 1, SVM-7) achieved the highest accuracy (96%), sensitivity (100%), NPV (100%), F1 score (0.92) and MCC (0.90) and a specificity of 95%, and PPV of 86%. Two single-sensor-based models ranked 11^th^ (Feature Subset 5 with head accelerometer sensor, SVM-4; and Feature Subset 6 with pelvis accelerometer sensor, SVM-4), achieving an accuracy of 88%, sensitivity 67%, specificity 95%, PPV 80%, NPV 90%, F1 score 0.73, and MCC 0.66. The twenty best models using feature selection were compared to the ten best models generated using all combinations of variables (AV) but no feature selection [17] (Table 4). The top fifteen models that used feature selection outperformed the best models that did not use feature selection.

**Table 3:** Feature-selection subsets used as inputs for faller classification models

Method	Feature-Selection Subset Output	Subset #
Relief-F	Insoles: Impulse I3, I6, and I7 Head: Maximum, mean, and standard deviation posterior acceleration Maximum, mean, and standard deviation anterior acceleration Mean superior acceleration	1
Relief-F	Pelvis: AP ratio of even to odd harmonics Maximum, mean, and standard deviation left acceleration Left Shank: ML Lyapunov exponent	2
Relief-F	Head: Vertical ratio of even to odd harmonics Mean and standard deviation posterior acceleration Pelvis: Maximum and standard deviation left acceleration	3
Relief-F	Insole: Impulse I1, I3, I4, I6, and I7 Pelvis: ML FFT first quartile AP Lyapunov exponent Maximum, mean, and standard deviation left acceleration	4
Relief-F	Head: ML and vertical FFT first quartile Vertical ratio of even to odd harmonics ML Lyapunov exponent Maximum, mean, and standard deviation right acceleration Maximum, mean, and standard deviation posterior acceleration Maximum, mean, and standard deviation anterior acceleration Maximum and mean superior acceleration	5
Relief-F	Pelvis: ML FFT first quartile AP ratio of even to odd harmonics AP, ML, and vertical Lyapunov exponent Maximum, mean, and standard deviation left acceleration Maximum and standard deviation inferior acceleration	6
Relief-F	Insole: Impulse I3, I6, and I7 Head: Maximum, mean, and standard deviation posterior acceleration Pelvis: ML FFT first quartile AP Lyapunov exponent Maximum and mean left acceleration	7
Relief-F	Pelvis: ML Lyapunov exponent Maximum, mean, and standard deviation left acceleration Left Shank: ML Lyapunov exponent Maximum and standard deviation left acceleration Maximum and standard deviation superior acceleration Right Shank: AP and vertical ratio of even to odd harmonics AP, ML, and vertical Lyapunov exponent Mean anterior acceleration	8
CFS/FCBF	Pelvis: Standard deviation left acceleration	9

*AP* anterior-posterior, *ML* medial-lateral, *FFT* fast Fourier transform

**Table 4:** Best twenty models using feature selection and best ten all variable (AV) models using a single 75:25 train:test stratified holdout. Feature subset numbers are defined in Table 3. For AV, feature set indicates the sensor and number of variables (in parentheses) in the subset

Method	Feature Set	Model^a^	Accuracy^b^ (%)	Sensitivity (%)	Specificity (%)	PPV (%)	NPV (%)	F1	MCC	SR
Relief-F	1	SVM-7	96.0 [80.4: 99.3]	100.0	94.7	85.7	100.0	0.923	0.901	33
Relief-F	1	SVM-6	92.0 [75.0: 97.8]	83.3	94.7	83.3	94.7	0.833	0.781	43
Relief-F	2	NN-15	88.0 [70.0: 95.8]	50.0	100.0	100.0	86.4	0.667	0.657	44
Relief-F	3	NN-21	88.0 [70.0: 95.8]	50.0	100.0	100.0	86.4	0.667	0.657	44
Relief-F	3	NN-23	88.0 [70.0: 95.8]	50.0	100.0	100.0	86.4	0.667	0.657	44
Relief-F	3	NN-25	88.0 [70.0: 95.8]	50.0	100.0	100.0	86.4	0.667	0.657	44
Relief-F	1	NN-21	88.0 [70.0: 95.8]	50.0	100.0	100.0	86.4	0.667	0.657	44
Relief-F	4	NN-9	88.0 [70.0: 95.8]	50.0	100.0	100.0	86.4	0.667	0.657	44
Relief-F	4	NN-21	88.0 [70.0: 95.8]	50.0	100.0	100.0	86.4	0.667	0.657	44
Relief-F	1	SVM-5	92.0 [75.0: 97.8]	100.0	89.5	75.0	100.0	0.857	0.819	52
Relief-F	5	SVM-4	88.0 [70.0: 95.8]	66.7	94.7	80.0	90.0	0.727	0.656	65
Relief-F	6	SVM-4	88.0 [70.0: 95.8]	66.7	94.7	80.0	90.0	0.727	0.656	65
Relief-F	7	NN-21	88.0 [70.0: 95.8]	66.7	94.7	80.0	90.0	0.727	0.656	65
Relief-F	3	SVM-3	88.0 [70.0: 95.8]	83.3	89.5	71.4	94.4	0.769	0.693	68
Relief-F	8	NB-Q	84.0 [65.3: 93.6]	83.3	84.2	62.5	94.1	0.714	0.618	102
AV	H(29)	SVM-4	84.0 [65.3: 93.6]	33.3	100.0	100.0	82.6	0.500	0.525	104
AV	I(30),H(29)	SVM-4	84.0 [65.3: 93.6]	33.3	100.0	100.0	82.6	0.500	0.525	104
AV	I(30),P(29), LS(29)	SVM-2	84.0 [65.3: 93.6]	33.3	100.0	100.0	82.6	0.500	0.525	104
AV	H(29),P(29), LS(29),RS(29)	NN-5	84.0 [65.3: 93.6]	33.3	100.0	100.0	82.6	0.500	0.525	104
CFS/FCBF	9	NN-8	84.0 [65.3: 93.6]	33.3	100.0	100.0	82.6	0.500	0.525	104
CFS/FCBF	9	NN-10	84.0 [65.3: 93.6]	33.3	100.0	100.0	82.6	0.500	0.525	104
AV	H(29)	SVM-2	84.0 [65.3: 93.6]	66.7	89.5	66.7	89.5	0.667	0.561	107
AV	I(30),P(29), LS(29),RS(29)	NB-Q	80.0 [60.9: 91.1]	83.3	78.9	55.6	93.8	0.667	0.554	120
AV	I(30),P(29)	SVM-2	84.0 [65.3: 93.6]	50.0	94.7	75.0	85.7	0.600	0.521	121
AV	I(30),H(29), P(29)	SVM-3	84.0 [65.3: 93.6]	50.0	94.7	75.0	85.7	0.600	0.521	121
AV	I(30),P(29)	NN-9	84.0 [65.3: 93.6]	50.0	94.7	75.0	85.7	0.600	0.521	121
AV	I(30),H(29), P(29),LS(29)	NN-20	84.0 [65.3: 93.6]	50.0	94.7	75.0	85.7	0.600	0.521	121
CFS/FCBF	9	NB-Q	76.0 [56.6: 88.5]	66.7	78.9	50.0	88.2	0.571	0.418	157
CFS/FCBF	9	SVM-2	80.0 [60.9: 91.1]	33.3	94.7	66.7	81.8	0.444	0.369	176
CFS/FCBF	9	SVM-3	80.0 [60.9: 91.1]	33.3	94.7	66.7	81.8	0.444	0.369	176

*AV* all variables, *I* pressure-sensing insole measures, *H* head accelerometer measures, *P* pelvis accelerometer measures, *LS* left shank accelerometer measures, *RS* right shank accelerometer measures, *NN* neural network, *NB* naïve Bayesian model, *SVM* support vector machine, *SR* summed rank

^a^NN-*a*, where *a* is the number of nodes in the hidden layer; SVM-*b*, where *b* is the polynomial degree; NB-Q is quadratic naïve Bayesian

^b^Accuracy [95% Confidence Interval]

**Fig. 3:**
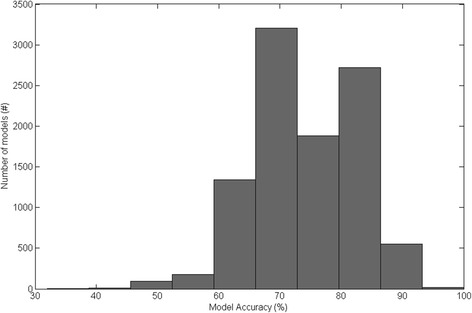
Histogram of RRS model accuracy for 10,000 randomizations of 75:25 train:test stratified holdouts for Feature Subset 1, SVM-7

The RRS model performance (Table 5) was lower than results from the single 75:25 train:test stratified holdout (Table 4) for all of the thirty evaluated models. The single 75:25 train:test stratified holdout used in the initial analysis resulted in model performance at the upper end of model performances observed over 10,000 randomizations. A histogram of RRS model accuracies is shown in Fig. 3, for Feature Subset 1, SVM-7, where the single holdout accuracy of 96% was one of the best accuracies. Similar to the single holdout results (Table 4), the top sixteen RRS models that used feature selection outperformed the best models that did not use feature selection (AV models). The top model (Feature Subset 1, SVM-7) from the single 75:25 train:test stratified holdout ranked second best after RRS, with 74% accuracy, 44% sensitivity, 83% specificity, 47% PPV, 83% NPV, 0.44 F1 score, and 0.29 MCC. The top RRS model (Feature Subset 9, SVM-2) achieved 78% accuracy, 26% sensitivity, 95% specificity, 65% PPV, 80% NPV, 0.36 F1 score, and 0.31 MCC.

**Table 5:** RRS model results for the best twenty models using feature selection and best ten all variable (AV) models. Feature subset numbers are defined in Table 3. For AV, feature set indicates the sensor and number of variables (in parentheses) in the subset. Results are mean ± standard deviation

Method	Feature Set	Model^a^	Accuracy (%)	Sensitivity (%)	Specificity (%)	PPV (%)	NPV (%)	F1	MCC	SR
CFS/FCBF	9	SVM-2	77.9 ± 4.8	26.4 ± 15.9	95.1 ± 5.2	64.6 ± 32.6	79.7 ± 3.5	0.355 ± 0.182	0.305 ± 0.202	55
Relief-F	1	SVM-7	74.0 ± 8.1	44.3 ± 20.2	83.3 ± 9.1	47.3 ± 20.1	82.8 ± 5.5	0.441 ± 0.173	0.286 ± 0.218	58
CFS/FCBF	9	SVM-3	78.0 ± 4.9	25.5 ± 15.6	95.5 ± 5.1	65.4 ± 33.5	79.5 ± 3.5	0.348 ± 0.183	0.304 ± 0.205	59
Relief-F	3	NN-21	75.3 ± 6.9	32.0 ± 20.4	89.7 ± 8.6	50.6 ± 29.7	80.1 ± 4.8	0.367 ± 0.203	0.259 ± 0.225	61
CFS/FCBF	9	NN-8	76.9 ± 6.0	27.6 ± 18.1	93.4 ± 8.0	60.2 ± 34.5	79.7 ± 4.0	0.349 ± 0.193	0.287 ± 0.212	62
Relief-F	1	SVM-5	73.6 ± 8.0	45.3 ± 20.1	82.6 ± 9.0	46.6 ± 19.1	82.9 ± 5.5	0.443 ± 0.169	0.285 ± 0.214	62
CFS/FCBF	9	NN-10	76.7 ± 6.0	28.2 ± 18.6	92.9 ± 7.9	58.3 ± 33.8	79.7 ± 4.1	0.351 ± 0.197	0.282 ± 0.214	64
Relief-F	5	SVM-4	74.6 ± 7.5	37.5 ± 20.2	86.3 ± 8.5	47.8 ± 23.9	81.6 ± 5.1	0.401 ± 0.188	0.262 ± 0.226	64
Relief-F	1	NN-21	76.0 ± 6.9	31.5 ± 20.2	90.1 ± 8.4	49.7 ± 30.1	80.9 ± 4.6	0.362 ± 0.205	0.258 ± 0.227	66
Relief-F	3	NN-25	75.3 ± 6.8	31.9 ± 20.6	89.7 ± 8.6	50.7 ± 29.5	80.1 ± 4.9	0.365 ± 0.202	0.257 ± 0.223	67
Relief-F	1	SVM-6	74.0 ± 7.6	38.5 ± 20.1	85.2 ± 8.7	46.5 ± 22.2	81.7 ± 5.2	0.402 ± 0.180	0.255 ± 0.219	71
Relief-F	3	NN-23	75.2 ± 6.8	31.9 ± 20.5	89.6 ± 8.5	50.3 ± 29.3	80.1 ± 4.8	0.365 ± 0.202	0.255 ± 0.225	74
Relief-F	2	NN-15	75.2 ± 7.1	30.3 ± 19.7	90.2 ± 8.8	50.9 ± 31.1	79.7 ± 4.7	0.356 ± 0.204	0.252 ± 0.230	76
Relief-F	8	NB-Q	68.3 ± 8.9	55.7 ± 20.6	72.5 ± 11.9	41.5 ± 13.6	83.5 ± 6.6	0.461 ± 0.140	0.264 ± 0.192	84
CFS/FCBF	9	NB-Q	70.9 ± 7.9	41.3 ± 22.4	80.7 ± 9.8	41.5 ± 20.0	80.9 ± 6.0	0.397 ± 0.184	0.221 ± 0.222	100
Relief-F	3	SVM-3	70.9 ± 8.0	37.9 ± 20.0	81.9 ± 9.6	42.2 ± 20.5	80.1 ± 5.5	0.381 ± 0.173	0.208 ± 0.214	102
AV	I(30),H(29), P(29)	SVM-3	75.5 ± 5.6	21.2 ± 16.2	93.6 ± 5.7	49.9 ± 35.2	78.2 ± 3.7	0.282 ± 0.195	0.207 ± 0.225	108
Relief-F	4	NN-9	73.4 ± 7.2	27.7 ± 19.5	88.7 ± 9.1	44.0 ± 29.8	78.8 ± 4.6	0.318 ± 0.201	0.196 ± 0.226	119
Relief-F	6	SVM-4	71.9 ± 7.3	31.7 ± 18.7	85.3 ± 8.2	42.2 ± 23.3	79.1 ± 4.8	0.346 ± 0.180	0.188 ± 0.218	120
Relief-F	4	NN-21	73.7 ± 6.8	25.3 ± 19.2	89.8 ± 8.6	43.0 ± 31.2	78.5 ± 4.4	0.298 ± 0.203	0.185 ± 0.225	126
Relief-F	7	NN-21	72.9 ± 6.9	25.8 ± 18.8	88.6 ± 9.0	41.7 ± 29.4	78.3 ± 4.4	0.298 ± 0.194	0.173 ± 0.218	139
AV	I(30),P(29), LS(29)	SVM-2	70.2 ± 7.1	30.8 ± 18.5	83.3 ± 8.7	38.4 ± 21.4	78.5 ± 4.7	0.326 ± 0.170	0.153 ± 0.204	139
AV	I(30),H(29)	SVM-4	74.2 ± 5.0	12.3 ± 13.2	93.7 ± 5.2	33.1 ± 35.8	77.2 ± 2.9	0.170 ± 0.175	0.087 ± 0.214	151
AV	H(29)	SVM-4	73.3 ± 5.8	16.1 ± 14.6	91.4 ± 6.4	35.1 ± 32.3	77.6 ± 3.3	0.209 ± 0.178	0.101 ± 0.215	153
AV	I(30),P(29)	SVM-2	67.6 ± 7.2	32.1 ± 18.4	79.4 ± 9.0	33.9 ± 17.6	78.0 ± 4.8	0.318 ± 0.159	0.117 ± 0.193	154
AV	I(30),P(29), LS(29),RS(29)	NB-Q	60.8 ± 9.2	37.6 ± 20.5	68.6 ± 11.7	28.3 ± 14.1	76.9 ± 6.4	0.314 ± 0.152	0.057 ± 0.203	171
AV	I(30),P(29)	NN-9	68.0 ± 8.3	24.4 ± 18.6	82.5 ± 10.6	31.4 ± 24.2	76.7 ± 4.8	0.258 ± 0.179	0.077 ± 0.216	178
AV	H(29)	SVM-2	67.8 ± 7.2	24.7 ± 17.3	81.4 ± 8.8	29.1 ± 19.7	77.5 ± 4.3	0.256 ± 0.163	0.063 ± 0.196	181
AV	I(30),H(29), P(29),LS(29)	NN-20	67.2 ± 7.9	21.0 ± 16.9	82.6 ± 10.4	27.8 ± 22.7	75.9 ± 4.3	0.226 ± 0.167	0.041 ± 0.199	190
AV	H(29),P(29), LS(29),RS(29)	NN-5	65.3 ± 8.2	16.5 ± 15.2	81.5 ± 11.0	22.3 ± 22.2	74.5 ± 4.0	0.177 ± 0.153	−0.021 ± 0.190	201

*AV* all variables, *I* pressure-sensing insole measures, *H* head accelerometer measures, *P* pelvis accelerometer measures, *LS* left shank accelerometer measures, *RS* right shank accelerometer measures, *NN* neural network, *NB* naïve Bayesian model, *SVM* support vector machine, *SR* summed rank

^a^NN-*a*, where *a* is the number of nodes in the hidden layer; SVM-*b*, where *b* is the polynomial degree; NB-Q is quadratic naïve Bayesian

## Discussion

The three feature selection techniques, CFS, FCBF, and Relief-F, successfully reduced the feature set from up to 146 features, derived from pressure-sensing insoles and four wearable accelerometers, to a viable set containing as few as one feature. Models derived using the reduced feature sets outperformed models derived using the full feature set when classifying fall risk, demonstrating the benefits of feature selection methods when creating faller classification models.

Relief-F feature selection performed well for the single 75:25 stratified holdout and RRS. A feature subset outputted by both CFS and FCBF feature selection techniques was used in the top RRS model. In other classification studies, CFS and FCBF provided the best feature subsets [16, 23]. However, these studies were not classifying elderly fall risk and instead classified human activities such as sitting, standing, and stair walking [23] or benchmark data sets that include healthcare diagnoses and census data [16]. Elderly fall risk is a complex classification problem where differences between fallers and non-fallers are often subtle and varied [24]. Relief-F feature selection has recognized strengths when dealing with noisy data sets and parameters with interdependencies [15], which may make this method suitable for elderly faller classification, in addition to CFS and FCBF feature selection techniques.

The best model (Feature Subset 1, SVM-7) for the single 75:25 stratified holdout and second best RRS model contained ten features: 3 pressure-sensing insole features and seven head accelerometer features. For the single 75:25 stratified holdout, this model achieved 96% accuracy, 0.92 F1 score, and 0.90 MCC. With 100% sensitivity, this model would be an excellent screening tool because all fallers would be identified. “Feature Subset 1, SVM-7” results were comparable to the best faller classification results in the literature: Caby et al. [8] with 100% accuracy and Giansanti et al. [25] with 97% accuracy. However, RRS model performance was lower, with an accuracy of 74%, F1 score of 0.44, and MCC of 0.29. This lower model performance is likely more indicative of future model performance given the large number of models trained with different data splits. With RRS analysis, this wearable sensor approach did not achieve 80% accuracy, which is often considered a threshold for good classification.

The pressure-sensing insole features in Feature Subset 1 were impulse measures I3, I6, and I7. I3 and I6 measure impulse during the second half of stance phase and I7 measures impulse during the entire stance phase. This indicates the importance of force magnitude and timing of force application during stance phase for faller identification, with fallers having lower I3, I6, and I7 impulse compared to non-fallers [24]. The lower impulse could indicate reduced force application due to muscle weakness, which is a fall risk factor [26, 27]. The head features were maximum, mean, and standard deviation for posterior and anterior acceleration, and mean superior acceleration. Head accelerations in the direction of progression was important for faller classification, with fallers having greater posterior and lower anterior acceleration compared to non-fallers [24].

Based on RRS model performances, the best model (Feature Subset 9, SVM-2) contained one feature from the posterior pelvis accelerometer: left acceleration standard deviation. This model achieved 78% accuracy, 26% sensitivity, 95% specificity, 65% PPV, 80% NPV, 0.36 F1 score, and 0.31 MCC. The posterior pelvis location allows unobtrusive and easy monitoring with a belt attached sensor or accelerometer-equipped smartphone, and high user acceptance was found in a 20-day case-study with a lower back sensor [28]. While this single-sensor RRS model ranked higher than the best multi-sensor RRS model, the single-sensor model had a much lower sensitivity 26% compared to 44% with the best multi-sensor model (Feature Subset 1, SVM-7). Given that the goal of the model is to identify fallers, the multi-sensor model with higher sensitivity (true positive rate), may be preferable even though the accuracy is lower (multi-sensor: 74%, single-sensor: 78%). The best RRS model sensitivity was 56% (Feature Subset 8, NB-Q) with accuracy 68%, F1 score 0.46, and MCC 0.26. The best single-sensor RRS model sensitivity was 41% (Feature Subset 9, posterior pelvis accelerometer only, NB-Q) with accuracy 71%, F1 score 0.40, and MCC 0.22.

Models with a feature subset performed better than models with a complete feature set, demonstrating the importance of including feature reduction when defining models for faller classification. Feature selection techniques removed irrelevant features and improved classification accuracy. Improved classification accuracy is one of the expected advantages of feature selection [3, 4, 23].

A stratified holdout was performed and confidence intervals calculated using the Wilson interval, as recommended by Shany et al. [29] in their recent review paper. Shany and colleagues recommended external validation as the optimal method for validating model performance, followed by holdout validation with confidence intervals computed, and finally cross validation, although it is currently not theoretically known whether cross-validation gives a better estimate of future model performance than simple holdout validation [29]. From our study, the results indicated that simple holdout validation may not accurately estimate future model performance, particularly when many models are investigated. Some accuracy confidence intervals did not include the average RRS model performance achieved across 10,000 stratified holdouts. For example, the accuracy for Feature Subset 1, SVM-7 was 96% with a 95% confidence interval of 80% to 99% for a single stratified holdout; however, the average RRS accuracy was 74%, which was 6% less than the lower confidence limit. With this older-adult gait dataset, a wide variance in model performance occurred with different train:test data divisions (Fig. 3). Therefore, cross-validation may be preferred over simple holdout validation for model development because cross-validation reduces the influence of the data partition on model performance. Furthermore, randomization of a large number of holdouts, such as the 10,000 randomizations performed in this study, may be preferred when evaluating model performance.

This study used retrospective fall occurrence as the criterion for classifying faller and non-faller status. While this is superior to using a clinical assessment based criterion [1], future studies should use prospective fall occurrence as the criterion for evaluating model classification performance. Retrospective fall occurrence is limited by inaccurate recall of falls and changes to gait patterns that occur between the fall and assessment, either in an attempt to increase stability or from fear of falling.

The use of the entire data set for feature selection allowed an analysis of consistent feature subsets across the different RRS data partitions and the comparison and recommendation of specific feature subsets. Feature selection on the entire data set may have overfit the feature subsets to the data set; therefore, the results should be confirmed with a new population sample to verify that the results are consistent for an older adult population that was not used for the feature selection process. While this study explored a large number of features (up to 146 features), other possible features could be included in future research. Additional accelerometer-based features from the literature, that were relevant to older adult fall risk, could be generated from phase-dependent local dynamic stability [30, 31], discrete wavelet transform [32, 33], sample entropy [5, 34], and power spectral density [35, 36]. Furthermore, this study examined features derived from a 7.62 m (25 ft) walking trial. This distance translates to clinical settings where the “25 ft Walk Test” [37, 38] could be performed; however, a longer walking trial may be more reflective of everyday walking for older adults. A study by Rispens et al. [34] found differences between treadmill-walking-based gait features and daily-life-walking-based gait features, with daily-life-based gait being more variable, less symmetric, and less stable compared to treadmill-based gait. Similar differences could be found when comparing lab-based, relatively short walking trials to daily-life walking. The 7.62 m walking distance may have affected MLE reliability, since stable MLE measures occurred after 35 strides in [39].

## Conclusion

Feature selection provided models with smaller feature sets and improved faller classification compared to faller classification without feature selection. CFS and FCBF provided the best feature subset for faller classification with a model based on one posterior pelvis accelerometer feature. However, better sensitivity was achieved by the second best model based on a Relief-F feature subset with three pressure-sensing insole features and seven head accelerometer features. Feature selection should be considered as an important step in faller classification using wearable sensors.

## Abbreviations

AP: Anterior-posterior
ASUFSR: Arizona State University Feature Selection Repository
AV: All variables
CFS: Correlation-based feature selection
CoP: Center of pressure
CoV: Coefficient of variation
FCBF: Fast correlation based filter
FFT: Fast Fourier transform
H: Head accelerometer measures
I: Pressure-sensing insole measures
LS: Left shank accelerometer measures
MCC: Matthew’s correlation coefficient
ML: Medial-lateral
MLE: Maximum Lyapunov exponent
NB: Naïve Bayes
NB-Q: Quadratic naïve Bayes
NN: Neural network
NPV: Negative predictive value
P: Pelvis accelerometer measures
PCA: Principal component analysis
PPV: Positive predictive value
REOH: Ratio of even to odd harmonics
RS: Right shank accelerometer measures
SR: Summed rank
SVM: Support vector machine
